# COVID-19 in Association With Development, Course, and Treatment of Systemic Autoimmune Rheumatic Diseases

**DOI:** 10.3389/fimmu.2020.611318

**Published:** 2021-01-26

**Authors:** Katja Lakota, Katja Perdan-Pirkmajer, Alojzija Hočevar, Snezna Sodin-Semrl, Žiga Rotar, Saša Čučnik, Polona Žigon

**Affiliations:** ^1^ Department of Rheumatology, University Medical Centre Ljubljana, Ljubljana, Slovenia; ^2^ Faculty of Mathematics, Natural Sciences and Information Technologies (FAMNIT), University of Primorska, Koper, Slovenia; ^3^ Faculty of Medicine, University of Ljubljana, Ljubljana, Slovenia; ^4^ Faculty of Pharmacy, University of Ljubljana, Ljubljana, Slovenia

**Keywords:** COVID-19, systemic autoimmune rheumatic diseases, autoantibodies, antiphospholipid antibodies, pediatric multisystem inflammatory syndrome in children, incidence, therapy, disease-modifying antirheumatic drugs

## Abstract

Autoimmune diseases and infections are often closely intertwined. Patients with autoimmune diseases are more susceptible to infections due to either active autoimmune disease or the medications used to treat them. Based on infections as environmental triggers of autoimmunity, an autoimmune response would also be expected in COVID-19. Although some studies have shown the occurance of autoantibodies and the possible development of autoimmune diseases after SARS-CoV-2 infection, current data suggest that the levels of autoantibodies following SARS-CoV-2 infection is comparable to that of some other known infections and that the autoantibodies might only be transient. The risk of SARS-CoV-2 infection in patients with a systemic autoimmune rheumatic disease (SARD) appears slightly higher compared to the general population and the course of COVID-19 disease does not seem to be very different, however, specific therapies such as glucocorticoids and anti-TNF might modulate the risk of hospitalization/death. Cytokine release syndrome is a severe complication in COVID-19. Many drugs used for the treatment of SARD are directly or indirectly targeting cytokines involved in the cytokine release syndrome, therefore it has been suggested that they could also be effective in COVID-19, but more evidence on the use of these medications for the treatment of COVID-19 is currently being collected.

## Introduction

Coronavirus disease 2019 (COVID-19) is a pandemic respiratory infectious disease caused by severe acute respiratory syndrome coronavirus 2 (SARS-CoV-2). Autoimmune diseases and infections are closely intertwined in several ways. Under normal conditions, the immune system exhibits tolerance to molecules recognized as “self,” and thus does not respond to elements that are expressed in endogenous tissues. Autoimmunity occurs when the self-tolerance is broken. Infections could act as an environmental trigger of autoimmune diseases, especially in genetically susceptible individuals. Several reports suggested that SARS-CoV-2 infection may be followed by an outbreak of autoimmune and autoinflammatory diseases (1). Next, an excessive inflammation observed in SARS-CoV-2 is mediated through cytokines that are drug targets in some SARD, and thus similar drugs were suggested to control excessive inflammatory reactions, e.g., the blockade of interleukin-6 (IL-6) signaling (2). Finally, a common point between infections and autoimmune diseases is the enhanced susceptibility of patients with SARD to infection as well as potentially altered course of infection due to immunomodulatory treatment (3).

## Risk of Developing SARD in Healthy Individuals Due to SARS-COV-2 Infection

Autoimmune diseases are a result of aberrant recognition of self-antigens by the immune system. Though the exact etiology of autoimmune diseases remains unknown, there are various factors which contribute to the emergence of an autoimmune disease, including the genetic predisposition and immune system dysregulation as well as the environmental triggers, such as infections. The three main mechanisms explain the development of autoimmunity: molecular mimicry, epitope spreading, and activation of neighboring antigenically non-specific immune cells, i.e., bystander activation (4). The breakdown of peripheral self‐tolerance can occur due to molecular mimicry, when a pathogenic antigen shares structural similarities with self-antigens. The pentapeptide sequence of the spike glycoprotein in SARS-CoV-2 which is responsible for virus entry into the cell, corresponds to the sequence of 24 surfactant-related human peptides, 13 of which lie on SARS-CoV-2 immunoreactive epitopes. Similarly, SARS-CoV-2 spike protein heptapeptides mimic 26 human proteome peptides, of which some encode human proteins involved in clinical complications of the COVID-19 disease (5). Epitope spreading facilitates the development of immune responses to endogenous epitopes secondary to the release of self-antigens during a chronic autoimmune or inflammatory response. The immune response to the pathogen or the pathogen itself may cause tissue lysis, and the released (neo-) antigens are taken up by the antigen-presenting cells, which provide a secondary immune response. The bystander activation is characterized by auto-reactive B and T cells undergoing activation in an antigen-independent manner, influencing the development of autoimmunity. The inflammatory environment damages the tissue to the point that non-specific antigens are exposed, while such an environment promotes immune cell activation. Additionally, a number of proteases that act in the inflammatory environment can process self-proteins, exposing normally invisible, non-dominant epitopes to the immune system (6).

The shared pathogenic mechanisms and clinical aspects between the hyper-inflammatory diseases and COVID-19 may suggest SARS-CoV-2 as a triggering factor for the development of a rapid autoimmune and/or autoinflammatory dysregulation, leading to severe interstitial pneumonia, in susceptible individuals (7). Accordingly, in a German study of 22 patients with acute respiratory failure due to SARS-CoV-2, radiographic and histomorphological similarities were found with acute onset of interstitial lung disease in connective tissue diseases. In the reported study, the presence of autoantibodies to nuclear antigens was found in 90% of patients admitted to the intensive care unit, as opposed to 36% of patients not requiring intensive care. This suggests that SARS-CoV-2 may induce autoimmunity, or COVID-19 may be more severe in patients who already have an autoimmune response, as we do not have a precise insight into when those autoantibodies first appeared (8). A similar, Chinese study of 21 patients admitted to the intensive care unit due to SARS-CoV-2 infection, confirmed the presence of autoantibodies to nuclear antigens in 50%, and antibodies to Ro antigen in 20% (9). Another study from Greece, included 29 patients without previously known SARD admitted to the intensive care unit due to SARS-CoV-2 infection, found antinuclear antibodies in 34%, anti-neutrophil cytoplasmic antibodies in 13%, anti-cardiolipin (aCL) in 24% and antibodies against β2-glycoprotein I (anti-β2-GPI) found in 34% of cases (10). As these autoantibodies carry pathogenic potential, the caution is needed, when considering the use of convalescent patient plasma for therapeutic purposes.

### Autoimmune Diseases Associated With COVID-19

#### Antiphospholipid Syndrome

Several groups have reported the occurrence of antiphospholipid antibodies (aPL) in patients with COVID-19 and suggested the possibility of SARS-CoV-2 virus induced antiphospholipid syndrome (APS) (3, 11, 12). APS is a syndrome clinically characterized by arterial, venous, or microvascular thrombotic events, and/or by pregnancy complications while serologically, it is defined by the persistent presence of aPL. The international classification criteria for definite APS updated in 2006 (13) include, in addition to clinical manifestations, the presence of three subgroups of aPL: lupus anticoagulants (LA), aCL, and anti-β2-GPI, which have to be positive twice at least 12 weeks apart. aPL can occur also transiently during various infections, including: skin infections (18%), human immunodeficiency virus infection (17%), pneumonia (14%), hepatitis C virus (13%), and urinary tract infections (10%) (14).

Coagulopathy and thrombotic events, including deep vein thrombosis, pulmonary embolism, and stroke are severe manifestations in critically ill patients with COVID-19. Elevated D-dimer represents the most significant abnormality of coagulation parameters in severe COVID-19 patients, and progressively increasing values can be used as a prognostic parameter indicating a worse outcome. Currently, the role of aPL in thrombotic complications in COVID-19 remains unclear. Similarly to severe coagulopathies associated with COVID-19, the subtype of APS patients may develop thromboses in several organs over a very short period (15). This is especially pronounced in the rare condition, called catastrophic APS, associated with a high mortality rate. Due to the similarity between the course of COVID-19 and APS, the hypothesis was made that SARS-CoV-2 infection could be a possible trigger for APS (**Table 1**). The analysis of 23 studies (together including a total of 250 patients) on aPL in COVID-19, showed that the presence of LA, aCL, and anti-β2-GPI was 64, 9, and 13%, respectively (16). IgM antibodies were the most common isotype. However, none of the included studies, reported retesting aPL after 12 weeks, thus it is not clear whether aPL presence in COVID-19 patients was transient or persistent. The only study in which aPL testing was repeated after 1 month, and in which also aPS/PT were measured, included 31 patients with COVID-19 (17). In this Belgium study, elevated aPL levels were confirmed in 23 (74%) patients, of which 21 (67%) patients had LA, seven aCL and aPS/PT, and three anti-β2-GPI. Nine of 10 retested LA positive patients were negative at second occasion. This observation supports the frequent single LA positivity during the acute phase of the COVID-19 infection; however, aPL were not clearly related to thrombotic complications. A recently published study of 122 COVID-19 patients determined the prevalence of IgG and IgM aCL in 13.4 and 2.7% and IgG and IgM anti-β2-GPI in 6.3 and 7.1% cases, which is significantly less frequent compared to the prevalence reported in APS patients (18). LA was detected in 22.2% of COVID-19 cases compared to 54.1% in APS. The authors did not confirm an association between arterial and venous thrombosis in COVID-19 and aPL. A recent study of eight types of aPL showed that a significant percentage (30-52%) of patients with COVID-19 became at least transiently positive for aPL and that these aPL are potentially pathogenic (19).

**Table 1:** Parallelism between coronavirus disease 2019 (COVID-19) and antiphospholipid syndrome (APS) (summarized after (11).

Antiphospholipid syndrome	COVID-19
Altered APTT, elevated D-dimer	Abnormal coagulation parameters in 69%: prolonged PT and altered APTT, elevated D-dimer, elevated FDP
Vascular thrombosis (≥ 1 clinically documented arterial, venous, or small vessel thrombosis)	Thromboembolic events occurred at a cumulate rate of 21%
Pulmonary complications	Large vessel thrombosis Pulmonary intravascular coagulopathy
Complement activation	Deposition of complement components C5b-9, C4d, and MASP2 in the lung and skin microvasculature
Disturbed cytokine balance and cytokine storm in CAPS	Increased release of inflammatory cytokines characteristic of a cytokine storm
Persistent presence of aPL (LA, aCL, anti-β2GPI) (i.e., determined 2x at 12-week intervals)	Transiently elevated aPL values (LA 64%, aCL 9%, anti-β2-GPI 13%) during the acute phase of the disease

*APTT, activated partial thromboplastin time; PT, prothrombin time; FDP, fibrin degradation products.

Another study investigated antigen specificity of aPL in COVID-19 and found that, contrary to APS, which is characterized by high aPL titers with specificity against domain 1 on β2-GPI, patients with COVID-19 exhibit low titers of anti-β2-GPI, with specificity against domains 4 and 5 (20).

The risk of recurrence of a thrombotic event in patients with APS is greatly increased in those who have multiple subtypes of aPL (LA, aCL, anti-β2-GPI, aPS/PT), i.e., double, triple positive patients. In patients with COVID-19, double or triple aPL positivity was rare and aPL positivity appears to be only transient. Additionally, the results were strongly influenced by pre-analytical factors, methodological issues, the heterogeneity of aPL, and finally, by insufficient standardization of diagnostic tests. In conclusion, there is currently no convincing evidence that aPL may play a relevant role in the hypercoagulable tendency of COVID-19.

#### Kawasaki Disease

**Figure 1:**
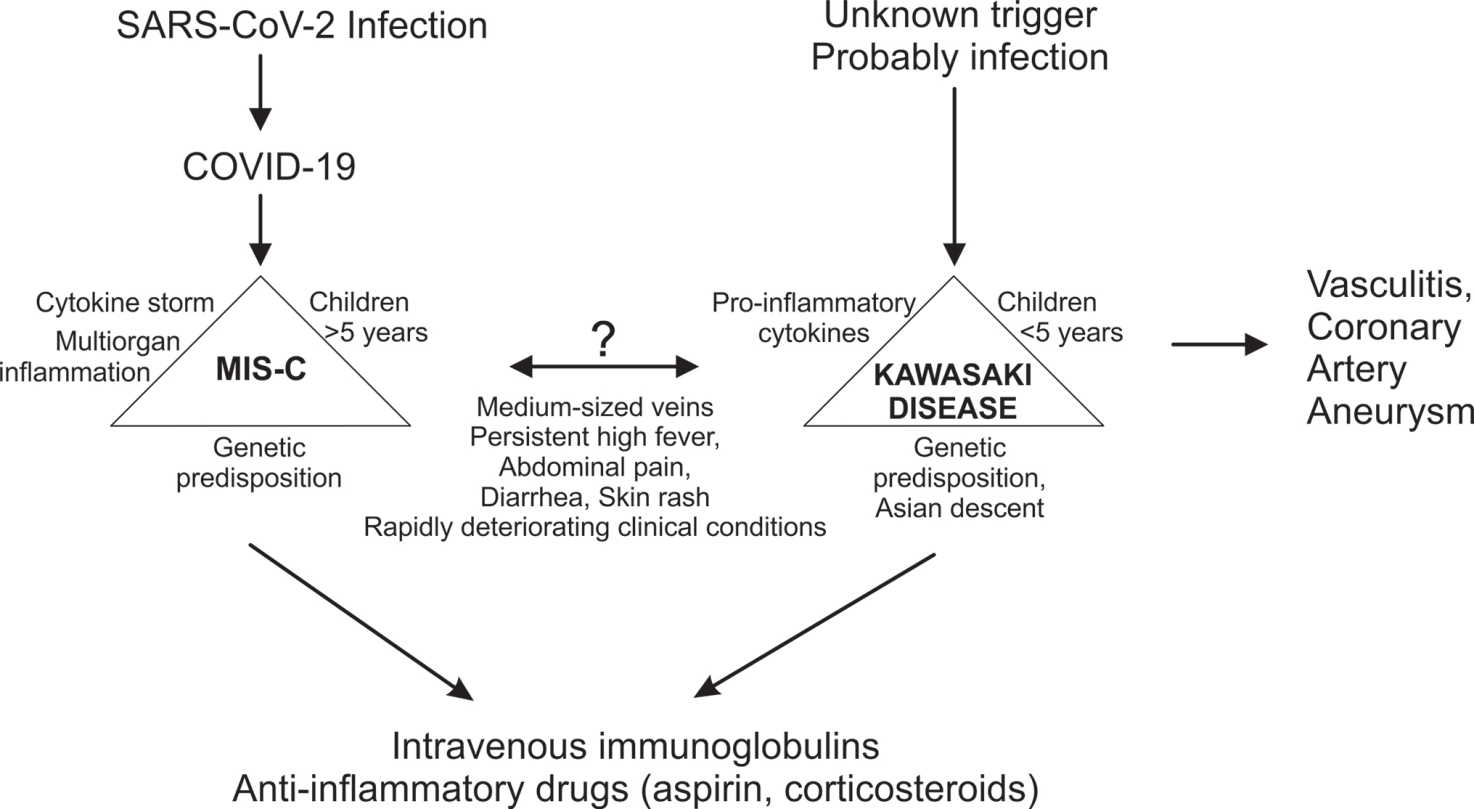
Comparison of multisystem inflammatory disease (MIS-C) related to coronavirus disease 2019 (COVID-19) and Kawasaki disease.

First publications on COVID-19 focused on adults, as SARS-CoV-2 infection appeared to be mostly mild in children. However, at the end of April 2020, both in North America and in Europe, the descriptions of a possible link between previous COVID-19 and occurrence of multisystem inflammatory syndrome in children (MIS-C) emerged. MIS-C related intense systemic inflammation often requires intensive treatment and can also lead to failure of one or more organ systems (21–23). The first definition of the case was proposed by the World Health Organization (WHO) in mid-May 2020 (24). MIS-C is thought to be caused by an incorrect immune system response to a specific trigger, which could include also the new coronavirus. MIS-C in association with SARS-CoV-2 manifests with fever, gastrointestinal, cardiovascular, mucocutaneous manifestations, and an increased release of cytokines and inflammatory markers (25). The syndrome is similar to Kawasaki disease (KD), which is thought to result from an excessive immune response to an infection in children with a genetic predisposition (**Figure 1**) (26). A hypersensitive reaction or disturbance in the immune response, probably triggered by infection, causes activation of the inflammatory process and lead to a systemic inflammation and vasculitis. KD is an acute systemic disease, associated with inflammation of medium-sized arteries, especially coronary arteries. Complications of KD are coronary artery dilatation and aneurysms. KD was first described in 1967 by Japanese pediatrician Tomisaku Kawasaki (27). KD is a rare disease, but together with IgA vasculitis it is one of the most typical forms of systemic vasculitis in children. It occurs all over the world, but most commonly in Japan. It usually affects younger children, and up to 85% of children with KD are younger than 5 years. Although cases of KD can occur at any time of the year, more cases seem to occur in late winter and spring.

Clinicians around the world observed several similarities between MIS-C and KD. Both diseases affect medium-sized vessels and manifest with persistently high fever, abdominal pain, diarrhea, skin rash, and rapidly deteriorating clinical condition. However, the main difference being that MIS-C most commonly affects children over five years of age. In addition, more gastrointestinal problems and more severe cardiovascular symptoms, as well as significantly higher inflammation markers were observed for MIS-C (25). The data also show that MIS-C is more common in countries with a higher COVID-19 load (3). A retrospective time-series analysis made in Paris, the French epicenter of the COVID-19 outbreak, estimated the incidence of KD in the last 15 years and confirmed a significant increase in the incidence of KD during the COVID-19 epidemic (28). An Italian study from the province of Bergamo, reported a 30-fold increase in the incidence of KD (0.3 per month before the epidemic, 10 per month at the time of the epidemic), with children diagnosed after the COVID-19 outbreak being older, had a higher rate of cardiac involvement, features of macrophage activation syndrome, and more patients more often required additional glucocorticoid treatment (29). A recently published review article covered 46 eligible case reports and case series, involving a total of 114 pediatric COVID-19 cases (23). Children had mostly had mild symptoms, including fever (64%), cough (35%), and rhinorrhea (16%) or no symptoms (15%). The main laboratory parameters were lymphopenia (33%), elevated D-dimer (52%), and C-reactive protein (40%). They identified 17 patients (15%) with MIS-C, with symptoms overlapping however distinct from KD. This review found that the majority of children with COVID-19 were generally less affected or asymptomatic, but infants might be seriously ill and older children may develop MIS-C with severe systemic impairment.

Recently, a systematic review on 39 observational studies has been published summarizing clinical presentations of 662 individuals with MIS-C (30). 71.0% of children (*n* = 470) were admitted to the intensive care unit, and 11 deaths (1.7%) were reported. Average length of hospital stay was 7.9 ± 0.6 days. Fever, abdominal pain, or diarrhea, and vomiting were the most common clinical presentation. As summarized by authors, children will typically show signs/symptoms of MIS-C 3 to 4 weeks after COVID-19 infection and many will progress rapidly into shock and cardiorespiratory failure. Prompt detection and medical treatment are of utmost importance.

## SARS-Cov-2 Infections in SARD Patients

### Risk of SARS-CoV-2 Infection in SARD

Patients with SARD are treated with immunomodulatory drugs that increase their susceptibility to infections (31). A recent analysis of 180 studies on the incidence of lower and upper respiratory tract infections in SARD confirmed the association between glucocorticoids and an increased incidence of viral respiratory tract infections. The use of Janus kinase (JAK) inhibitors, especially in high doses, as well as tumor necrosis factor (TNFi) and interleukin-17 (IL-17i) inhibitors has been associated with mild viral respiratory infections, while non-steroidal anti-inflammatory drugs, hydroxychloroquine, methotrexate, and some other drugs have not been associated with an increased risk of respiratory infections (32). It is therefore reasonable to be cautious in regard to COVID-19 related morbidity in patients with SARD.

Four studies from Italy and a similar study from Spain investigated the incidence of SARS-CoV-2 infections in more than 4,300 SARD patients and reported a similar incidence (0.2 and 0.6% in all studies) of laboratory-proven diagnoses of COVID-19 among SARD patients in comparison to a locally and a time-comparable population without SARD (33–37). Similar findings are reported from Hong Kong (38) (**Table 2**). One of the studies from Italy reports significantly increased prevalence of COVID-19 in SARD patients (0.7%) as compared to the general population, (0.35%; OR: 1.31, 95% CI 1.05–3.52) (39) and two phone surveys found increased prevalence in SLE patients (2.5%) (45) and in patients with large-vessel vasculitis (2.5%) (44), but the latter study did not comment their findings compared to the general population. In a retrospective study of hospital PCR-confirmed COVID-19 cases, the authors report on 0.76% prevalence among 26,131 rheumatologic patients from 7 centers from Spain (ranged from 0.23 to 1.24% in different regions) as compared to a global prevalence of 0.58% (46). Recently published review analyzed 62 observational studies with a total of 319,025 autoimmune patients (including inflammatory bowel disease, autoimmune hepatitis, and autoimmune skin disease), 878 of whom had COVID-19. They reported an overall 0.27% prevalence of COVID-19, the highest observed in the SLE/SSc/SjS group (47). In the same paper meta-analysis of six case-controlled studies demonstrated that the risk of COVID-19 in rheumatic diseases (256 cases/29,578 included) was higher than in the control patients (OR: 1.6 95 CI%: 1.13 to 2.25) and meta- regression analysis showed a higher prevalence of COVID-19 in studies having high proportion of patients receiving glucocorticoids.

**Table 2:** Studies analyzing incidence of coronavirus disease 2019 (COVID-19) infection in systemic autoimmune rheumatic disease (SARD) and case descriptions.

Study	Cohort	Result
**Emmi et al. (** 33 **)**	Italy; 458 SARD pts. (117 SLE, 37 SjS, 17 APS, 64 arthritis, 149 vasculitis, 15 familial Mediterranean fever, 9 recurrent idiopathic pericarditis, 22 others)*43% were taking TNFi, 55% glucocorticoids*	7 tested pts., 1 positive—prevalence 0.22% (0.01–1.21%), comparable to that observed in the general population of Tuscany [0.20% (0.20–0.21%)].The patient was admitted to intensive care unit.
**Zen et al. (** 34 **)**	Italy; 916 pts. (397 SLE, 182 ANCA associated vasculitis, 176 SSc, 111 RA, 50 idiopathic inflammatory myopathy) *36% were taking antimalarial drug*	65 tested pts; 2 (0.21%) positive, a proportion similar to that observed in the general population of the Veneto region.
**Favalli et al. (** 36 **)**	Italy; 955 pts. (531 RA, 203 PsA, 181 SpA, and 40 of CTD/vasculitides/autoinflammatory diseases) *55% were taking TNFi*	The incidence of confirmed COVID-19 is consistent with the general population (0.62 *vs*. 0.66%). None of the pts. developed severe respiratory involvement (three were hospitalized) or died.
**Ferri et al. (** 39 **)**	Italy; 1641 pts (695 RA, 208 PsA, 35 AS, 438 SSc, 76 SLE, 64 UCTD, 18 SjS, 19 PM/DM, 88 other)	11 confirmed COVID-19 (0.67%) significantly higher than general Italian population (0.35%) (p=0.03). Two patients developed severe COVID-19, of them one died.
**Michelena et al. (** 35 **)**	Spain; 959 SARD pts. (RA, PsA, SpA, JIA, autoinflammatory syndromes)—all pts. receiving bDMARD or tsDMARD *74% were taking TNFi*	COVID-19 incidence rates were very similar to that of the general population [(0.48% (95% CI 0.09 to 0.87%)] and [0.58% (95% CI 0.56 to 0.60%)]. All 11 pts. recovered, 6 were admitted to hospital, one required intensive care unit.
**Quartuccio et al. (** 37 **)**	Italy; 1051 SARD pts. (362 RA, 275 PsA, 176 AS or non-radiograph. spondyloarthritis, 74 systemic vasculitides, 38 SLE, 19 other chronic inflammatory diseases) *59% were taking TNFi*	3.8/1000 (95% CI 1.5–9.7/1,000) pts. positive by PCR for COVID-1, compared to 2/1,000, 95% CI 1.9–2.1/1,000 in general population (p=0.33). 3/4 pts. were admitted in hospital, none to intensive care unit, all favorable outcome.
**Favalli et al. (** 40 **)**	Italy; 530 pts. (49% RA, 36% SpA/PsA, 10% JIA, 3% CTD, 1 patient with sarcoidosis) *53% were taking TNFi*	3 pts. confirmed COVID-19, one required hospitalization, none died; 10 pts. reported contact with COVID-19 positive-none developed infection.
**Monti et al. (** 41 **)**	Italy; 320 pts. (57% RA, 43% SpA) treated with bDMARD or tsDMARD; *52% treated with TNFi*	4 pts. confirmed COVID-19, four with clinical symptoms and five with contacts, but no symptoms. No death or severe COVID-19.Chronic arthritis pts. treated with bDMARDs or tsDMARDs do not seem to be at increased risk of respiratory or life-threatening complications from SARS-CoV-2 compared with the general population. No relapses of rheumatic disease observed.
**Conticini et al. (** 42 **)**	Italy; 859 SARD pts. (411 RA, 192 PsA, 131 AS, 54 SSc, 6 SLE, 26 vasculitides, 8 sarcoidosis, and 9 other) treated with stable and full dosage of bDMARDs or tsDMARD*41% treated with TNFi*	2 pts. diagnosed with COVID-19, one hospitalized and discharged after 3 days, other remained asymptomatic. Pts. treated with bDMARDs or tsDMARDs did not develop life-threatening complications from COVID-19.
**Jovani et al. (** 43 **)**	Spain; 1,037 rheumatic disease pts taking bDMARD/tsDMARD (exact diagnoses and therapy not reported)	3 pts. were hospitalized due COVID19, none required oxygen supply. 2 pts. developed acute pyelonephritis.
**So et al. (** 38 **)**	Hong Kong; 39,835 rheumatologic disease pts. (exact diagnoses and therapy for cohort not reported)	Incidence 0.0126% of COVID-19 confirmed in rheumatologic disease pts., compared to 0.0142% in the general population All pts. made uneventful recovery without complications or flare of underlying diseases.
**Tomelleri et al. (** 44 **)**	Italy; 162 large vessel vasculitis (67 Takayasu arteritis, 95 giant cell arteritis) 62% taking glucocorticoids, 32% taking tocilizumab	4 pts. confirmed COVID-19 (incidence 2.5%), 12 pts. with clinical symptoms, 2 pts. were hospitalized, oxygen not needed. None of pts experienced relapse of LVV (until April).
**Bozzalla Cassione et al. (** 45 **)**	Italy; 165 SLE 77% taking hydoxychloroquine, 56% taking prednisolone	2.5% Incidence higher as compared to the general population (0.76–0.47%). 4 pts. confirmed COVID-19, 8 with clinical symptoms and 7 with contacts no symptoms. 1 pt. required intensive care unit.

Each study is described by month of paper publication; diagnoses in study cohort are reported (ANCA, anti-neutrophil cytoplasmic antibodies; APS, antiphospholipid syndrome; AS, ankylosing spondylitis; CTD, connective tissue disease; b/tsDMARDs, biological/targeted synthetic disease-modifying antirheumatic drugs; JIA, juvenile idiopathic arthritis; PsA, psoriatic arthritis; Pts., patients; RA, rheumatoid arthritis; SjS, Sjögren syndrome; SLE, systemic lupus erythematosus; PM/DM, polymyositis/dermatomyositis; SpA, spondyloarthritis; SSc, systemic sclerosis; TNFi, TNF inhibitor) and most prevalent therapy taken in study population are described.

The data published so far suggest that the incidence of COVID-19 in SARD patients might be slightly higher than in the general population, thus only studies including a high number of patients are able to find this. Also, patients with autoimmune diseases may have been tested earlier and more frequently compared to the general population, but it should also be noted that SARD patients suspected of having an increased risk may have followed the recommendations for social distancing more carefully than the general population.

### Course of COVID-19 in SARD

**Figure 2:**
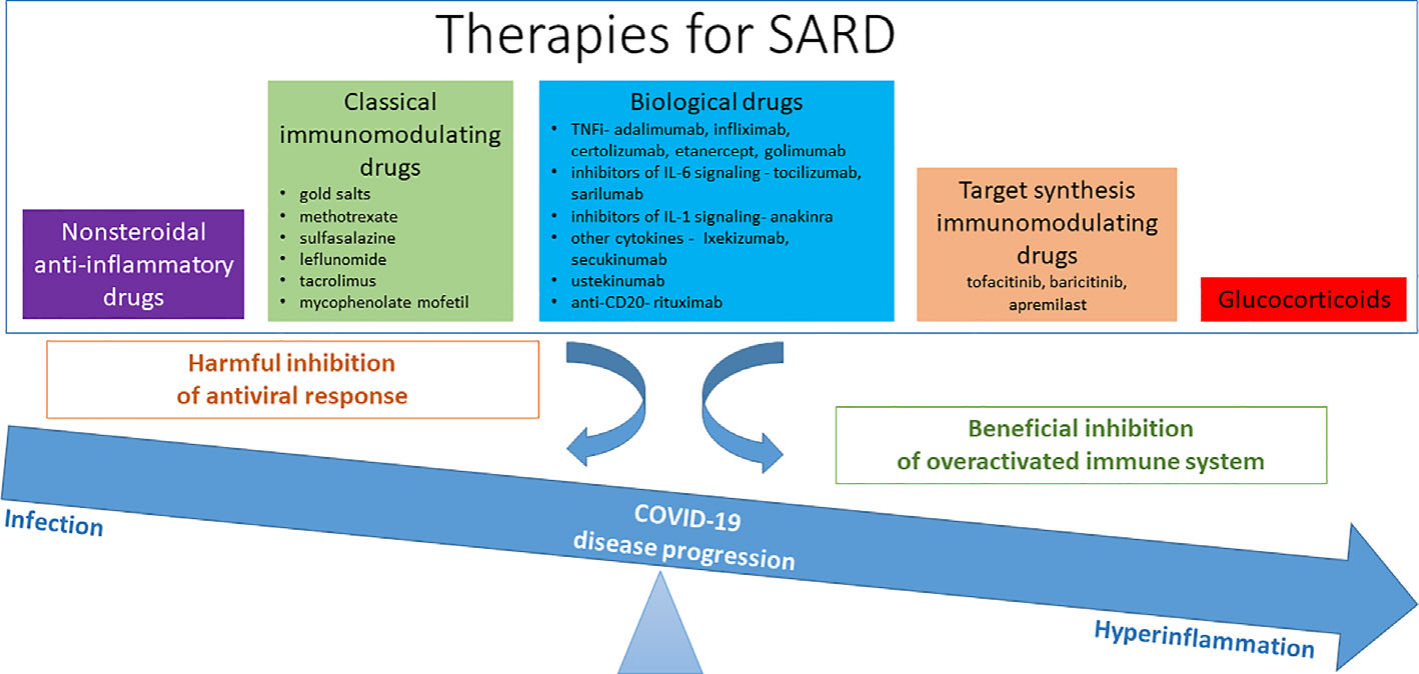
Therapy for systemic autoimmune rheumatic disease (SARD) and their possible effect/role in coronavirus disease 2019 (COVID-19).

Isolated cases of less pathogenic coronaviruses, which can cause pneumonia in immunocompromised patients, have been described, therefore concerns about SARS-CoV-2 in patients with SARD is reasonable (48). On the other hand, already established immunomodulatory and anti-inflammatory therapies of patients with SARD, could affect their immune status long-term, even after immediate discontinuation of therapy. Reducing the body’s response to infection and lowering antiviral defense could have a negative effect in the early stages of the COVID-19. However, in later stages it could also favor and possibly prevent cytokine storm complications in the severe course of COVID-19 (**Figure 2**).

Among studies, mentioned in analyses of COVID-19 incidence, 13 of 24 COVID-19 positive SARD patients were reported to be hospitalized, all with favorable outcome (33–37). Another three studies from Italian rheumatologists comprising altogether 1,709 patients with SARD, found 9 SARS-CoV-2 infected patients (40–42). Among these, only one patient required a short-term hospitalization. Similarly, in a Spanish study, 3 out of 1,037 patients on biological or targeted synthetic immunomodulatory therapy were hospitalized, none of whom required oxygen therapy (43) (**Table 2**).

In studies comparing the symptoms and the course of COVID-19 in patients with SARD *versus* patients without SARD, the proportion of hospitalizations, the risk of a severe course of COVID-19, the time from onset of symptoms to intubation, the duration of intensive care were all comparable (49–53) (**Table 3**). In patients with inflammatory rheumatic disease, also hospitalizations (56, 62), more severe course (50) and mortality (62) were associated with similar comorbid factors than in the general population—i.e., age, gender, hypertension, and diabetes (**Table 3**). Two studies report on patients with connective tissue disease/autoimmune systemic conditions having a higher risk of hospitalization and severe COVID-19 than patients with inflammatory arthritis (50, 61). A study from the UK, that analyzed factors related to the mortality of more than 5,000 patients hospitalized for COVID-19 found, that patients with autoimmune diseases (rheumatoid arthritis, psoriasis, systemic lupus erythematosus) had a 1.23 times higher risk [HR 1.23 (95% CI: 1.12–1.35)] for death, though no specific SARD diagnosis stood out. On the other hand, risks due to age, gender, obesity, diabetes, respiratory diseases (excluding asthma), the risk in patients after organ transplantation and hematological malignancies were all higher compared to having SARD (38). Likewise, 103 COVID-19 patients with rheumatoid arthritis or spondyloarthritis from New York, had a comparable hospitalization rate and frequency of deaths rates as COVID-19 patients from general population (56).

**Table 3:** Studies of coronavirus disease 2019 (COVID-19) course and outcomes in rheumatic disease patients.

Study	Cohort	Result
**Case control studies comparing rheumatic COVID-19 positive *versus* non-rheumatic COVID-19 positive**		
**D’Silva et al. (** 54 **)**	US; 52 rheumatic pts. (19 RA, 10 SLE, 7 PMR, 15 other) positive for COVID-19 and *104 age and sex matched non-rheumatic COVID-19 positive comparators*	Similar symptoms, manifestations, and outcome, similar % of hospitalization (44% SARD *vs*. 42% control), similar mortality; more often intensive care admission/mechanic ventilation is SARD (48 *vs*. 18%).
**Pablos et al. (** 50 **)**	Spain; 228 rheumatic pts. with confirmed COVID-19 (60% inflammatory arthritis (RA; PsA, SpA), 40% CTD (SLE, SjS, SSc, vasculitis), and *228 age and sex matched non-rheumatic controls*	Risk for severe COVID was not increased in rheumatic group; risk was increased with age and male sex and in CTD pts. *versus* inflammatory arthritis (OR 1.82 95% CI 1.02–3.30) Therapy was not associated with risk for severe COVID-19.
**Ansarin et al. (** 51 **)**	Iran; 30 COVID-19 positive with autoimmune disease (14 RA, 4 SSc, 3 SLE, 9 other) *versus 381 COVID-19 positive immunomodulatory drug naive pts.*	The frequency of clinical manifestations (malaise, dyspnea, myalgia, anosmia, and taste loss) were significantly higher in pts. treated with immunomodulatory drugs compared with immunomodulatory drugs-naïve pts. No significant differences were observed in the admission level, time interval between the onset of symptoms and intubation, duration of intubation, duration of admission in ICU, and number of deceased pts. in the two groups.
**Fredi et al. (** 53 **)**	Italy; 26 rheumatic musculoskeletal disease pts. *versus* 62 controls	No significant differences between cases and controls in duration of COVID-19 symptoms before admission, duration of stay in hospital, or the local chest X-ray scoring system.
**Ye et al. (** 52 **)**	China, 21 pts. COVID-19 with rheumatic disease (8 RA, 4 SLE, 3 SjS, 2 CTD, 2 PMR, 1 JIA, 2 AS) *versus 2,301 COVID-19 positive pts.*	No statistical difference in hospitalization time, significantly more respiratory failure (38 vs. 10%) and no different mortality rate. 4 rheumatic pts. undergone flare of rheumatic disease (pain in joints, back pain, rash, hemolytic anemia, muscle aches).
**Studies including only autoimmune COVID-19 positive group**		
**Gianfrancesco et al. (** 55 **)**	COVID-19 Global Rheumatology Alliance registry; 600 COVID-19 positive in people with rheumatic disease (38% RA, 14% SLE, 12% PsA)	46% confirmed cases were hospitalized; 9% died. The use of non-steroidal anti-inflammatory drugs, antimalarials, conventional DMARD alone, or in combination with biologics/JAK inhibitors was not associated with hospitalization due to COVID-19. Glucocorticoid exposure of ≥10 mg/day was associated with a higher odds of hospitalization and anti-TNF with a decreased odds of hospitalization in pts. with rheumatic disease.
**Haberman et al. (** 56 **)**	US; 103 inflammatory arthritis (RA, SpA, PsA) pts. with confirmed/highly susceptive COVID-19 – (80 confirmed, 23 highly susceptive for COVID-19)	26% pts. required hospitalization, 4% died. Pts. needing hospitalization were older, with hypertension, COBP. Pts. on glucocorticoids more likely to be admitted to hospital, while those on anti-cytokine therapy no association was found.
**Winthrop et al. (** 57 **)**	US, Canada; 77 cases using immunomodulatory drugs (24% RA, 6% UC, 6% sarcoidosis)	81% pts. were hospitalized, 35% required mechanical ventilation; 11% died. Pts. with anti-TNF had lowest hospitalization rate, admittance to intensive care unit and none died.
**Fredi et al. (** 53 **)**	Italy, 1,525 rheumatic and musculoskeletal disease pts.	65 confirmed COVID-19, 52 suggestive pts.; of confirmed cases 47 (72%) admitted to hospital and 12 pts. died (deceased older than survivors).
**Scire et al. (** 58 **)**	Italy; 232 pts. (34% RA, 26% SpA 21% CTD, 11% vasculitis)	70% hospitalized, 19% death Clinical presentation of COVID-19 was typical, with systemic symptoms (fever and asthenia) and respiratory symptoms (64% pneumonia), males worse prognosis. Immunomodulatory treatments were not significantly associated with an increased risk of intensive care unit admission/mechanical ventilation/death.
**Flood et al. (** 59 **)**	Ireland; 40 community acquired COVID-19 in inflammatory rheumatic diseased (14 RA, 4 SLE, 4 AS, 2 JIA, 2 SjS, 5 CTD, 2 other)	15% of hospitalization; hospitalization less likely in those on bDMARD (0% hospitalized pts. used bDMARD *vs*. 47% non-hospitalized pts. using bDMARD).
**Mathian et al. (** 60 **)**	17 SLE with SARS-CoV-2; all pts. but one with quiescent SLE, median treatment on HCQ time 7.5 years; 12 pts. receiving prednisolone	Viral pneumonia in 13 (76%) pts., complications due to respiratory failure in 11 (65%), acute respiratory distress syndrome in 5 (29%), 3 acute renal failure, 2 requiring hemodialysis. Five (36%) discharged from the hospital, two (50%) remained hospitalized, and two (14%) died. Except one pt. with active tenosynovitis at the onset of SARS-CoV-2 infection, none of the pts. showed clinical signs of lupus. Hydroxychlorokine does not seem to prevent COVID-19, at least its severe forms in pts. with SLE.
**Freites Nunez et al. (** 61 **)**	Spain; 123 pts. with AIRD (50 RA, 18 AS, 6 PsA, 8 inflammatory polyarthritis, 8 SLE, 6 PMR, 6 MCTD, 9 SjS, 12 other) and symptomatic COVID-19	54 (44%) pts. were hospitalized, 20 developed relevant complications, 12 patients died (PCR test not performed in 75% non-admitted and 19% admitted patients). After adjusting for comorbidities and age and sex only systemic autoimmune condition (opposed to inflammatory arthritis OR 3.55; 95% CI 1.3–9.7) and age (OR 1.08; 95% CI 1.04–1.13) remained significant contributors to hospitalization, no effects of SARD therapy observed.
**Studies of pts. with rheumatic diseases hospitalized due COVID-19**		
**Santos et al. (** 62 **)**	Spain; 38 COVID-19 pts. with rheumatic and musculoskeletal disease admitted to hospital (16 RA, 8 PMR, 5 SLE, 2 AS, 7 other)	10 pts. died of COVID-19, death associated with age, hypertension, diabetes, with moderate/high index of rheumatic disease activity; no association with background therapy (glucocorticoids, MTX) or symptoms before admission in deceased and survivors.
**Zhao et al. (** 63 **)**	China; 29 rheumatic disease pts. with COVID-19 that were hospitalized (15 RA, 5 SLE, 9 other)	Lower prevalence of mechanical ventilation needed in rheumatic pts. than in D’Silva paper (54) (here 5%); 1/29 died (3%).
**Sanchez-Piedra et al. (** 64 **)**	Spain registry of SARD; 41 pts. had COVID-19 (21 with RA, 12 SpA, 8 other)	28 (68%) pts. hospitalized and 3 (7%) patient died; in general population hospitalization rate 53% and mortality rate 12%.

APS, antiphospholipid syndrome; AS, ankylosing spondylitis; CTD, connective tissue disease; DMARDs, disease-modifying antirheumatic drugs; JIA, juvenile idiopathic arthritis; PMR, polymyalgia rheumatica; PsA, psoriatic arthritis; Pts., patients; RA, rheumatoid arthritis; SjS, Sjögren syndrome; SLE, systemic lupus erythematosus; SpA, spondyloarthritis; SSc, systemic sclerosis; UC, ulcerative colitis).

Impact of SARD therapy on the course of the COVID-19 disease was analyzed in 600 SARD patients with COVID-19 included in the COVID-19 Global Rheumatology Alliance database, which currently includes over 6,000 patients from 40 countries. The use of NSAIDs, antimalarials, conventional synthetic immunomodulatory drugs alone or in combination with biologics and JAK inhibitors has not been associated with an increased likelihood of hospitalization due to COVID-19. The report even suggests the patients treated with TNFi were less likely to be hospitalized. The use of glucocorticoids (prednisolone ≥ 10 mg QD) was associated with an increased likelihood of hospitalization (55). Similar findings regarding glucocorticoids have been found in a study of 103 patients with inflammatory arthritis (65), while a Spanish study of 228 patients with inflammatory rheumatic disease found no association between the severity of COVID-19 and therapy (50). Immunomodulatory treatments were similarly not significantly associated with an increased risk of intensive care unit admission/mechanical ventilation/death in an Italian study of 232 RMDs patients with proven SARS-CoV-2 infection who used corticosteroids (58) and in a Spanish study of 123 patients with AIRD and COVID-19, although in the latter study data on glucocorticoid doses was not reported (61).

A systematic review comprising 65 observational studies published by the end of July 2020, assessed 2,766 patients with different autoimmune diseases (hepatic, skin, inflammatory bowel disease, rheumatic diseases, and systemic autoimmune diseases) and diagnosed with COVID-19, identified rates of hospitalization (rheumatic diseases 0.54; p=0.29; SLE/SSc/SjS 0.32; p=0.04) and mortality rates (rheumatic diseases 0.113; p<0.001; SLE/SSc/SjS 0.069; p<0.001). However, hospitalisation and death rates in the normal population have not been reported and compared. Importantly, patients receiving anti-TNF monotherapy tended to have lower hospitalisation and mortality rates than patients receiving non-TNF-directed monotherapy. The same study reports meta-analysis of 3 case-control studies of rheumatic disease patients showed no differences in hospitalization (OR 0.875; p=0.44), and death rates for rheumatic disease patients (OR 1.434; p=0.13) (47). Age, comorbidities, glucocorticoids, and conventional synthetic immunomodulatory drugs alone or in combination with biologics and JAK inhibitors increased the risk for severe outcomes, whereas targeted synthetic immunomodulatory drugs or biologics monotherapy, particularly anti-TNF agents, were associated with a lower risk of hospitalization and death.

### Course of SARD During the Infection With SARS-CoV-2

Only few studies specifically mention the course of SARD during COVID-19 infection itself. Among the 17 patients with systemic lupus erythematosus in remission who developed COVID-19, there was no relapse of the disease at the time of infection (60). No relapses were observed also among 320 patients with chronic arthritis, of which 4 had confirmed COVID-19 (41). In the study including 21 patients COVID-19 positive rheumatic patients 4 undergone flares including joint pain, back pain, muscle aches, rash, and hemolytic anemia that were treated with drugs (52).

### Recommendations for the Treatment of SARD During COVID-19 Pandemic

Following recommendations of German Society for Rheumatology, published in April (66), EULAR (European League Against Rheumatism) published provisional recommendations for the management of SARD in the context of SARS-CoV-2 (67). Their principal message is that there is no indication that patients with SARD have higher risk of contracting the virus or that worse disease course is to be expected. There are a few more highlights such as that rheumatologist should definitely be involved in discussions on whether to stop or start SARD treatment while managing COVID-19 infection in a SARD patient.

There are 13 recommendations that cover 4 themes: general measures and prevention of SARS-CoV-2 infection; the management of SARD patients during the pandemic; the management of SARD patients who have COVID-19; and the prevention of other pulmonary infections in SARD patients.

Their main messages are the following:

1. We should follow the regular guidelines in our country; if a patient with SARD does not have symptoms of COVID-19, continue regular treatments.
2. SARD patients should avoid visits to the hospital or to the office; remote monitoring *via* the telephone should be used when possible;
3. In the absence of known exposure, in the absence of COVID-19 infection, the EULAR panel felt very strongly about the importance of continuing rheumatic disease treatments.
4. If patients test positive, then four recommendations cover what to do, such as continuing use of SARD treatments, but in the case of glucocorticoids this should be the lowest possible dose necessary. Mild, worsening, and significant COVID 19 symptoms are clearly defined. In cases of mild symptoms, the recommendation is to “decide on a case-by-case basis.” If a patient’s symptoms worsen, then we should »seek expert advice immediately and follow local treatment recommendations«. Regardless of COVID-19 severity, anti-malarial therapies may be continued.

On the whole, the EULAR recommendations are fairly similar to those already released by the American College of Rheumatology. There are differences such that the European recommendations advise on immunizations and pneumonia prophylaxis, saying that all patients without COVID-19 symptoms should make sure they are up to date with any recommended vaccinations. Another difference is that the ACR recommendations could potentially be updated monthly if the evidence arrives to allow that, while EULAR expecting to update its recommendations every 3 months (68).

## Cytokine Release Syndrome and the Use of Antirheumatics

COVID-19 is a global health emergency of international concern and poses a particular threat to elderly or to patients with specific chronic diseases and/or multimorbidity. Increased susceptibility to infection and poorer initial antiviral immune response are factors that determine whether the infection will remain asymptomatic or clinical symptoms of fever, cough, shortness of breath, headache, or in more severe cases pneumonia and an excessive systemic immune response will occur. An excessive immune response, which has been previously described also in the Spanish flu in 1919, as well as in SARS and MERS, was always associated with a high mortality rate. The syndrome of cytokine release, in severe forms called a cytokine storm, manifests as a syndrome of acute respiratory distress, and multiorgan failure, and is described up to 5–20% of patients with COVID-19. Cytokines lead to extravasation of immune cells into tissues, destabilization and apoptosis of endothelium and epithelium, damage of capillaries, and multi-organ failure (69). In COVID-19, prognostic factors of severe disease course include elevated CRP, IL-6, ferritin and D-dimer, lymphopenia, decreased monocyte and T cell counts. Increased secretion of several cytokines, including IL-2, IL-7, IL-6, IL-10 G-CSF, IP10, MCP-1, MIP-1, TNF was found. Several reports confirm that elevated serum levels of these cytokines correlate with the severity of COVID-19 (70). A detailed analysis of 1,484 patients with COVID-19 showed that TNF and IL-6 levels, independently correlated with disease outcome (71). A cytokine storm, with the activation of macrophages, can also occur after the application of certain treatments, such as, posttransplant Graft-*versus*-host disease, and upon chimeric antigen receptor (CAR) T-cell therapy (72). This also sets the medical grounds where the gained experience in treatment for the current epidemic comes from. As many medications used for SARD are directly or indirectly targeting cytokines involved in cytokine release syndrome, it has been suggested that may also benefit in COVID-19. The use of drugs such as tocilizumab, methotrexate, hydroxychloroquine, tofacitinib, sulfasalazine on patient derived cell cultures, mouse disease models, and tissue biopsies from patients with rheumatoid arthritis has shown that these drugs inhibit the secretion of cytokines greatly elevated in COVID-19 (73).

### Targeted Anti-Cytokine Therapies Neutralize Individual Mediators of Inflammation

IL-6 is a pleiotropic cytokine that plays a key role in the cytokine storm. The monoclonal antibodies tocilizumab and sarilumab target the IL-6 receptor and are approved for the treatment of rheumatoid arthritis, the former also for giant cell arteritis and for CAR-T cell therapy-induced cytokine release syndrome. While first reports indicated the successful use of anti-IL-6 drugs for the treatment of COVID-19 (74), two randomized studies (one with tocilizumab and the other with sarilumab) failed to demonstrate the positive effects of the aforementioned treatment (75). However, a USA retrospective analysis of electronic health records among 5,776 patients with COVID-19 cytokine storm between March and April 2020 reported that the combination of corticosteroids with tocilizumab showed superior survival outcome when compared with standard-of-care treatment and treatment with corticosteroids alone or in combination with anakinra (76).

Anakinra is a recombinant IL-1 receptor antagonist used to treat certain autoinflammatory diseases. Data from a randomized phase 3 study in patients with sepsis suggest better survival in patients with macrophage activation syndrome during anakinra treatment (77). Currently, there is an ongoing study in patients with COVID-19 where anakinra is co-administered with an anti-interferon γ antibody (emapalumab).

Monoclonal antibodies directed against TNF were the first biological therapy in the treatment of SARD. While developing anti-tumor drugs, it has been found that TNFi successfully inhibit the expression of IL-6, GM-CSF, and IL-1. Although TNFi increase the risk of viral infections, studies in SARD do not indicate an increased number of COVID-19 infections, among TNFi-treated patients, on the contrary, they have even suggested protective effects (55). A randomized study CATALYST is currently underway to evaluate the efficacy of TNFi and anti-GM-CSF in COVID-19.

### Drugs That Inhibit the Action of Several Cytokines

JAK inhibitors are newer target synthetic immunomodulatory drugs that inhibit the intracellular signal transduction of various inflammatory cytokines, including interferon type I, and IFNγ, and thus inhibit the primary antiviral response. They also inhibit tyrosine kinases that are involved in the intracellular viral transport and endocytosis into epithelial cells. Baricitinib and tofacitinib harbor FDA/EMA warning box of increased risk of thrombotic complications (78). A hypercoagulable state is also present in COVID-19 patients, however there are currently 37 registered clinical studies with JAK inhibitors in association with SARS-CoV-2 treatment (79). Based on the ACTT-2 trial, which compared the combination of remdesivir and barcitinib to remdesivir alone and showed significantly shorter time to recovery, lower odds to patient’s condition progressing and higher odds for clinical improvement with the use of baricitinib, the FDA granted emergency approval for this treatment in mid-November 2020 (80).

### Chloroquine/Hydroxychloroquine

Antimalarials are used in the treatment of various SARD. *In vitro* data suggest potential efficacy, as COVID-19 therapy, since antimalarials alter endosomal pH, affect the transport of SARS-CoV-2 to endolysosomes, alter glycosylation of the ACE2 receptor, and severely inhibit virus replication (81). On the other hand, antimalarials lower type I interferons, which are the first line antiviral defense. Following initial enthusiasm for antimalarials in COVID-19, further studies in COVID-19-infected people reported mixed results (regarding the incidence of infection, the likelihood of developing respiratory distress syndrome, or death in COVID-19 patients). A randomized study showed that hydroxychloroquine did not prevent COVID-19 when used up to 4 days after contact with an infected person (82). In patients with systemic lupus erythematosus treated with hydroxychloroquine, antimalarials did not prevent the disease (60, 83) or decreased hospitalization rate or admission to intensive care unit (84). Perhaps it is also the consequence of serum concentrations in the treatment of systemic lupus erythematosus that are around 0.5 mg/L, while antiviral activity requires significantly higher serum concentrations (4mg/L) (85). The adverse events of high doses of the drug on cardiac function, with prolongation of the QT interval, are an additional burden to patients. However, there are over 100 ongoing registered clinical studies with antimalarials associated with COVID-19 treatment (79).

### Glucocorticoids

Glucocorticoids inhibit inflammation and have an immunosuppressive effect by acting directly on the expression of inflammatory cytokines, while reducing the proliferation and differentiation of lymphocytes and macrophages and thus may increase susceptibility to COVID-19. In patients with SARS and MERS, glucocorticoids were used for prevention and treatment of acute respiratory distress syndrome, but it was shown that they did not reduce mortality. Contrary, glucocorticoids slowed virus clearance and had some other side effects, like psychosis, diabetes, and avascular necrosis (86). A randomized study in COVID-19 also yielded mixed results based on whether patients were already receiving oxygen at the time of randomization (87). In recent meta-analysis of clinical trials of critically ill COVID-19 patients showed that the administration of systemic corticosteroids led to lower 28-day all-cause mortality compared to usual care (88) and retrospective analysis of health records of 5776 patients with COVID-19 cytokine storm shows corticosteroid improved hospital survival (76). Until recently there was little evidence of a beneficial effect of glucocorticoids, especially if they were prescribed in high doses in acute respiratory distress syndrome (89), however in September 2020, European Medicine Agency endorsed the use of dexamethasone in COVID-19 patients on oxygen and mechanical ventilation based on results of RECOVERY study where dexametasone reduced mortality (90). The decision was supported by additional a meta-analysis conducted by the WHO which looked at data from seven clinical studies investigating the use of corticosteroids for the treatment of patients with COVID-19.

## Conclusion

**Figure 3:**
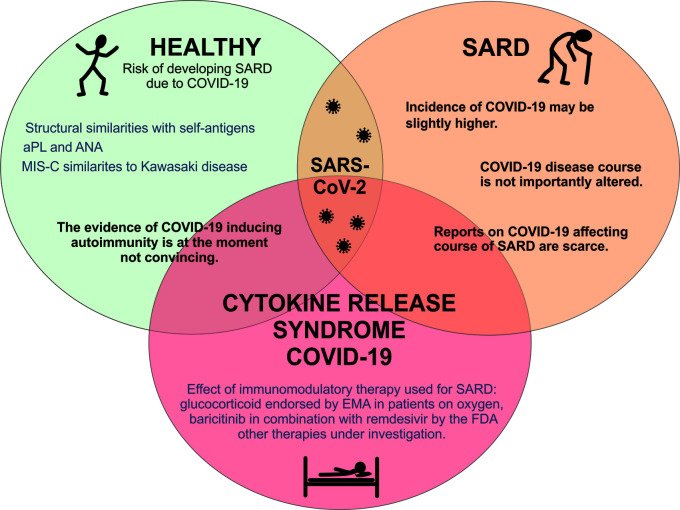
Graphical summary of SARS-CoV-2 infection and systemic autoimmune diseases. APS, antiphospholipid syndrome; EMA, European Medicine Agency; MIS-C, multisystem inflammatory syndrome in children; SARD, systemic autoimmune rheumatic diseases; SARS CoV-2, severe acute respiratory syndrome coronavirus 2; COVID-19, coronavirus disease.

In conclusion, this paper discussed three key areas of COVID-19 in the relation to SARD, as shown in the graphical summary (**Figure 3**). First, although infections can trigger autoimmune diseases by various mechanisms and some autoantibodies (such as aPL and antinuclear antibodies) have been detected in SARS-CoV-2 infected patients, the evidence for autoimmune inducibility of COVID 19 is currently not convincing.

Second, the risk of COVID-19 infection in patients with SARD in some studies parallels general population but in others a slight increase as compared to general population is shown. The course of COVID-19 disease in SARD patients (hospitalization, admission to the intensive care unit, death) does not appear to differ significantly from the general population, although comparative studies suggest that it also depends on age, comorbidities, and therapy used for SARD treatment. To date, there have been no comprehensive reports regarding relapses of SARD in COVID-19 patients.

Third, the use of glucocorticoids in COVID-19 patients treated with oxygen or mechanical ventilation has been endorsed by the EMA, as has the use of baricitinib in combination with remdesivir by the FDA, while evidence that any other immunomodulatory drug (conventional or targeted synthetic or biological) used to treat SARD would protect against the more severe course of COVID-19 is lacking.

## Author Contributions

KL and PZ conceptualized the paper, reviewed the literature, and wrote the first edition of the manuscript. KP-P, AH, SS-S, ŽR, and SČ contributed to the manuscript with their expertise, read, edited, and approved the submitted version. All authors contributed to the article and approved the submitted version.

## Funding

The study was supported by the Slovenian Research Agency ARRS, with the post-doctoral project Z3-9261 and the National Research Program #P3-0314.

## Conflict of Interest

The authors declare that the research was conducted in the absence of any commercial or financial relationships that could be construed as a potential conflict of interest.
